# Research Progress Concerning Dual Blockade of Lymphocyte-Activation Gene 3 and Programmed Death-1/Programmed Death-1 Ligand-1 Blockade in Cancer Immunotherapy: Preclinical and Clinical Evidence of This Potentially More Effective Immunotherapy Strategy

**DOI:** 10.3389/fimmu.2020.563258

**Published:** 2021-01-08

**Authors:** Yihang Qi, Li Chen, Qiang Liu, Xiangyi Kong, Yi Fang, Jing Wang

**Affiliations:** Department of Breast Surgical Oncology, National Cancer Center/National Clinical Research Center for Cancer/Cancer Hospital, Chinese Academy of Medical Sciences and Peking Union Medical College, Beijing, China

**Keywords:** cancer, immunotherapy, PD-L1, PD-1, LAG3, CD223

## Abstract

Although various immunotherapies have exerted promising effects on cancer treatment, many patients with cancer continue to exhibit poor responses. Because of its negative regulatory effects on T cells and its biological functions related to immune and inflammatory responses, there has been considerable emphasis on a protein-coding gene named lymphocyte-activation gene 3 (LAG3). Recently, evidence demonstrated marked synergy in its targeted therapy with programmed death-1 and programmed death-1 ligand-1 (PD-1/PD-L1) blockade, and a variety of LAG3 targeted agents are in clinical trials, indicating the important role of LAG3 in immunotherapy. This mini-review discusses preclinical and clinical studies investigating PD-1 pathway blockade in combination with LAG3 inhibition as a potentially more effective immunotherapy strategy for further development in the clinic. This strategy might provide a new approach for the design of more effective and precise cancer immune checkpoint therapies.

## Introduction

In the past decade, many studies have investigated immunotherapy for various cancers, and the benefits of programmed death-1 (PD-1) and programmed death-1 ligand-1 (PD-L1) inhibitor therapies have been demonstrated (1, 2). Nevertheless, the objective response rate was 13%–56%, while the complete response rate was 1%–16%; these findings suggested that the effects of PD-1/PD-L1 targeted immunotherapy were less robust than originally reported (3–8). Consequently, there is an urgent need for novel agents, either as new immune targets or to facilitate and enhance conventional treatments.

Lymphocyte-activation gene 3 (LAG3), also known as CD233, is a protein-coding gene currently under clinical investigation as a promising inhibitory receptor, following programmed death-1 (PD-1)/programmed death-1 ligand-1 (PD-L1) and cytotoxic T-lymphocyte-associated protein 4 (CTLA-4), due to its negative regulatory effects on T cells and its biological functions related to immune and inflammatory responses (9, 10). Recently, there has been substantial preclinical and clinical evidence to support the use of PD-1 pathway blockade in combination with LAG3 inhibition as a potential effective immunotherapy strategy. This mini-review briefly summarizes the structure, isoforms, ligands, signaling, and immune-related functions and roles of LAG3 in immunotherapy, and discusses the basic and clinical research related to the effects of dual LAG3 and PD-1/PD-L1 blockade. Additionally, it demonstrates how LAG3 might function as an important component in anti-tumor immunotherapy. This comprehensive review focuses on this potential effective immunotherapy strategy to guide the development of cancer immunotherapy.

## PD-1/PD-L1 Structures and Functions

PD-1, also known as CD279, is a coinhibitory receptor expressed on the surface of antigen-stimulated T cells (2, 11, 12). PD-1 interacts with two ligands, PD-L1 (CD274) and PD-L2 (CD273). PD-L2 is expressed on cells such as macrophages, dendritic cells, and mast cells; PD-L1 is expressed on hematopoietic cells including T cells, B cells, macrophages, dendritic cells, and mast cells. PD-L1 is also expressed on various non-hematopoietic cells in healthy tissues, including vascular endothelial cells, astrocytes, keratinocytes, placenta syncytiotrophoblast cells, corneal epithelial and endothelial cells, and pancreatic islet cells (11, 13). PD-L1 is widely considered the dominant inhibitory ligand of PD-1 on T cells in the human tumor microenvironment. PD-1 and PD-L1 are type I transmembrane proteins, which belong to the immunoglobulin (Ig) superfamily. PD-1 contains an Ig-V-like extracellular transmembrane domain and a cytoplasmic domain, which harbors two tyrosine-based signaling motifs (12, 14, 15). In contrast, PD-L1 consists of an Ig-V and Ig-C-like extracellular transmembrane domain and a short cytoplasmic domain, which does not contain any canonical signaling motifs (15). When PD-1 is engaged by PD-L1, T-cell proliferation, survival, cytokine production, and other functions are inhibited (11).

## LAG3 Structure and Ligands

As a member of the Ig superfamily, LAG3 encodes a 498-amino acid membrane protein, which contains four extracellular immunoglobulin superfamily-like domains (D1–D4) (16). The structure of LAG3 has been partially elucidated. First, it includes a 30-amino acid proline-rich loop in D1, which mediates the interaction of LAG3 with major histocompatibility complex class II (MHC-II) molecules (17). Second, a long connecting peptide is present in the D4 transmembrane domain, which makes LAG3 susceptible to cleavage by transmembrane metalloproteases. Third, the cytoplasmic domain consists of three conserved motifs: a serine-based motif that acts as a protein kinase C substrate, a “KIEELE” motif with an important lysine residue that is crucial for downregulation of T-cell functions (18, 19), and an “EP” motif consisting of repetitive glutamic acid and proline dipeptides. Nevertheless, the downstream signaling pathways and the functions of each motif remain poorly understood. The first reported canonical LAG3 ligand was MHC-II, followed by other potential ligands: galectin-3 (20, 21), liver sinusoidal endothelial cell lectin (LSECtin) (22), fibrinogen-like protein 1 (23), and pre-formed fibrils of α-synuclein (24).

## s-LAG3 Structure and Ligands

The soluble form of LAG3, s-LAG3, is a 54-kDa fragment cleaved from the D4 domain connecting peptide released from cells by shedding at the cell surface (25). s-LAG3 is mediated by disinterring and metalloproteinase domain-containing protein 10 and 17 (ADAM10 and ADAM17), which also induce the cleavage of other immune receptors including VEGFR2, TIM3, CD62L, and TNFα (23). sLAG3 had an adjuvant effect on a DNA tumor vaccine, which maintained most 1-year old BALB-neuT transgenic mice tumor free, with markedly extended disease-free survival and reduced mammary adenocarcinoma tumor multiplicity. Although s-LAG3 was not the intended target of clinical trials using LAG3-specific monoclonal antibodies, it might be informative to bear in mind the role of sLAG3 in T cell mediated immunity. s-LAG3 was thought to bind only to MHC-II molecules present in lipid raft microdomains on a minor subset of antigen presenting cells. s-LAG3 might be an early T-cell-specific biomarker for type 1 diabetes onset. Furthermore, reduced s-LAG3, suggesting decreased LAG3 cleavage, was associated with inhibition of type 1 diabetes after treatment (26). s-LAG3 appears to be rapidly degraded and loses the ability to bind to MHC-II molecules as a result of not being dimeric (27). However, other studies suggested that s-LAG3 is important for interactions with dendritic cells (DCs) (28). Although the function of s-LAG3 is unclear, several studies suggest s-LAG3 might be a valuable circulating biomarker for cancer prognosis. For example, in patients with estrogen or progesterone receptor-positive breast cancer, a high level of serum s-LAG3 correlated with favorable disease-free survival, metastasis-free survival, and overall disease-specific survival (29). Similar findings were recently observed in a study of patients with gastric cancer (30). These data provide evidence to support further investigations of s-LAG3 as a predictive or prognostic biomarker for use with LAG3-targeted and other cancer therapies.

## Functions of LAG3 in Cancer-Related Immune Regulation and Dysfunction

LAG3 is widely expressed on the membranes of various immune cells, including T cells (CD4+/CD8+ T cells), regulatory T cells, B cells, and natural killer cells (31). Furthermore, LAG3 is stored in lysosomes, facilitating its rapid appearance on the cell surface following T-cell activation (32). LAG3 principally interacts with MHC-II molecules expressed on the surfaces of antigen presenting cells and tumor cells (33, 34) (**Figure S1**).

In the tumor microenvironment, LAG3 has a negative regulatory effect on T-cell responses, resulting in T-cell dysfunction (**Figure S2**). LAG3 inhibits CD4+, CD8+, and natural killer T-cell proliferation; cytokine production; and cytolytic function (35). When LAG3 is blocked, its suppressive influence on activated effector CD4+ T cells is inhibited through conformation-dependent recognition of stable peptide/MHC-II complexes (36), especially for regulatory T cells (e.g., both activated induced CD4+ FoxP3+ regulatory T cells and natural regulatory T cells). For non-regulatory CD4+ T cells, ectopic expression of LAG3 also confers suppressive activity (36). Additionally, expression of LAG3 on CD8+ T and natural killer cells symbolizes a dysfunctional profile (37). Finally, LAG3 inhibits the functions of antigen-presenting cells (e.g., dendritic cells) by blocking and interfering with their maturation when bound to LAG3+ regulatory T cells (38).

## Putative Mechanisms of LAG3 Immunotherapy Strategy

The extracellular component of LAG3 is structurally similar to CD4 with four immunoglobulin superfamily-like domains (D1–D4) and an additional proline-rich loop in the D1 domain required for its binding to MHC-II, which can be aberrantly expressed on tumors. Although MHC-II was reported to be the canonical ligand for LAG3, studies using a rat mAb (clone C9B7W) to mouse LAG3 that binds to the D2 domain without disrupting LAG3/MHC-II interactions reported that C9B7W improved anti-tumor responses associated with enhanced T cell proliferation and effector function (relative to responses elicited by an isotype-matched control antibody) (39, 40). Furthermore, C9B7W does not block the LAG3/MHC-II interaction but is a potent inhibitor of LAG3 function *in vitro* and *in vivo* (41, 42). These results indicate that binding to MHC-II may be dispensable for the functions of LAG3 and suggests other ligands may exist, especially in the context of the role of LAG3 on CD8+ tumor-infiltrating lymphocytes (TILs). LAG3 is highly expressed on TILs in a variety of cancers (43, 44).

**Figure 1:**
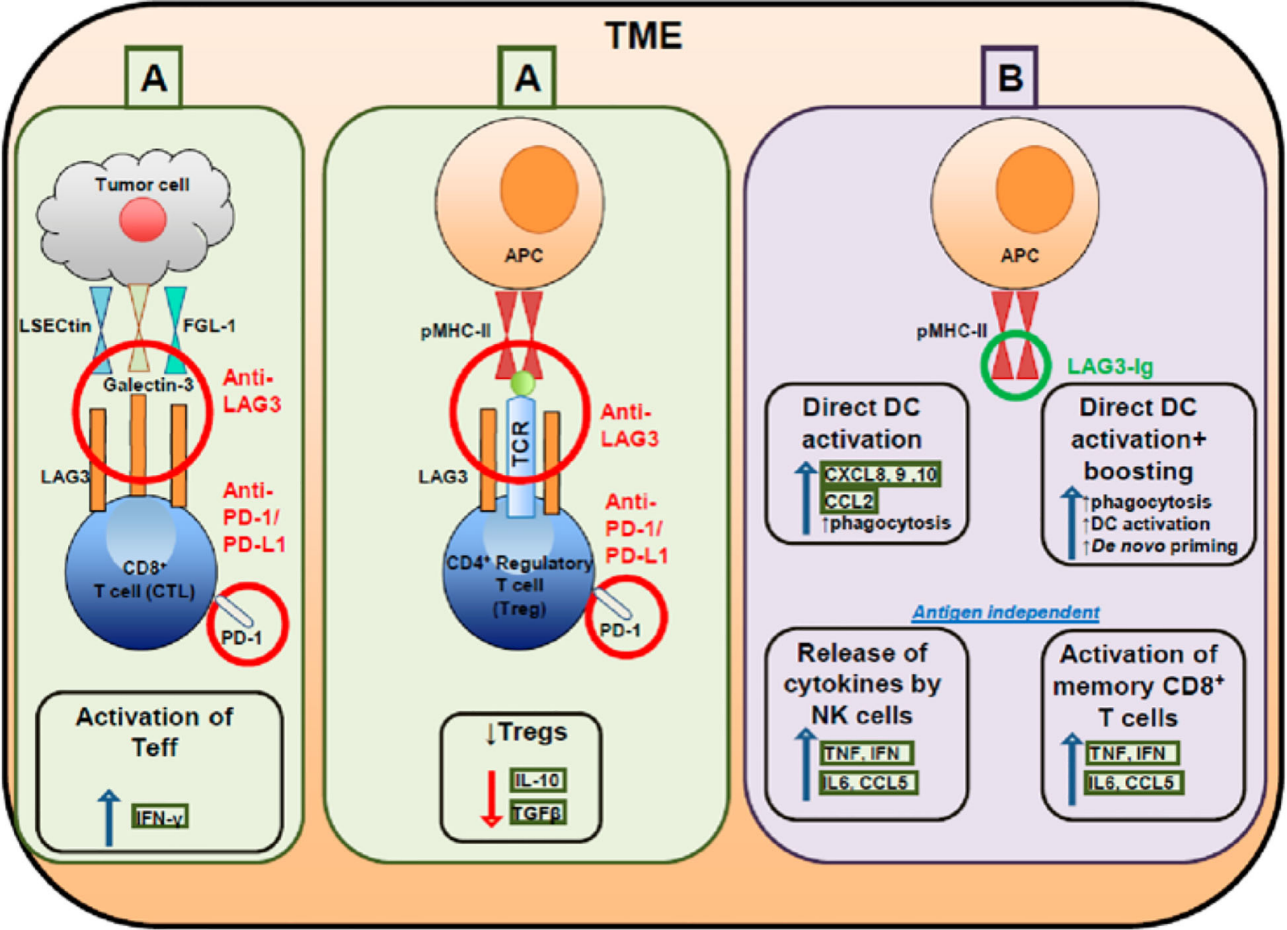
Targeting effector and regulatory T cells with LAG3 antagonistic antibodies **(A)** and activating antigen presenting cells with soluble LAG3 immunoglobulin (Ig) **(B)**.

**Figure 2:**
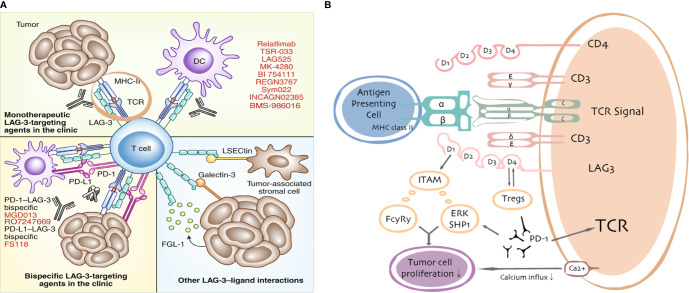
LAG3/ligand interactions. Current targeting strategies in the clinic **(A)** and putative interactions between LAG3 and PD-1/PD-L1 **(B)**.

LAG3 is associated with the exhaustion program of dysfunctional CD8+ TILs, demonstrated by the marked reduction of cytokine production, cytolytic activity, and cell proliferation (9). Co-expression of LAG3 and PD-1 correlated with intratumoral T cell dysfunction in patients (45). Furthermore, the co-expression of PD-1 and LAG3 decreases levels of the inflammatory cytokines IFN-γ and TNF; however, blockade of LAG3 and PD-1 improved cell proliferation and increased cytokine production in antigen-specific CD8+ TILs (46). LAG3 is constitutively expressed by a subset of thymus-derived regulatory T cells (Treg cells) and the co-expression of LAG3 and integrin CD49b identified type 1 Treg cells that produce IL-10 (47). LAG3 is involved in the ITAM inhibitory signaling pathway and inhibits DC maturation. LAG3 engagement with MHC-II induces an ITAM-mediated inhibitory signaling pathway, which involves the FcγRγ and ERK-mediated recruitment of SHP-1, and suppresses dendritic cell maturation and immunostimulatory capacity (38, 48, 49). LAG3 negatively regulates the CD3/TCR activation pathway and inhibits cell proliferation and cytokine secretion in response to CD3 signaling. LAG3 is specifically co-localized with the CD3-TCR complex and downregulates CD3/TCR complex expression by CD4/CD8 molecules, which results in the functional unresponsiveness of T cells (48, 50–52). Furthermore, LAG3 is also a ligand for MHC-II molecules and co-caps with the CD3/TCR complex to inhibit cell proliferation and cytokine secretion in response to CD3 signaling (53) (**Figures 1**, **2B**).

## Preclinical Evidence for the LAG3 Immunotherapy Strategy

In a BALB/c mouse model of triple negative breast cancer (TNBC), Du et al. found that tumor growth was significantly inhibited upon treatment with LAG3 and PD-1 dual blockade; the final tumor weights and volumes of this group were markedly reduced compared with those of the LAG3 and PD-1 single blockade groups, as well as the control group (54). In other tumor models, synergism involving PD1 and LAG3 was reported, suggesting the dual blockade of LAG3 and PD1 has sufficient efficacy for the treatment of various tumors. Seng et al. found that LAG3 was coexpressed with PD1 on tumor-infiltrating CD4+ and CD8+ T cells in melanoma (B16-F10), colon adenocarcinoma (MC38), and fibrosarcoma (Sa1N) tumors (55). In mice with MC38 and Sa1N tumors, LAG3 monotherapy was generally ineffective with very limited tumor clearance and slight reduction of tumor growth, whereas dual blockade of LAG3 and PD1 synergistically restricted tumor growth and achieved tumor clearance in 80% of mice compared with 40% remission in mice receiving anti-PD1 monotherapy (55). In the Sa1N tumor model, dual blockade of LAG3 and PD1 achieved tumor clearance in 70% of mice compared with 20% survival in mice receiving anti-PD1 monotherapy. In a study by Huang et al., C57BL/6 mice with ovarian tumors derived from ID8 cells were randomly divided into groups; each group was administered anti-PD-1 treatment, anti-LAG3 treatment, or combined anti-PD-1 and anti-LAG3 treatment. The results showed that dual blockade of PD1 and LAG3 synergistically enhanced anti-tumor immunity by inhibiting tumor growth and enhanced infiltration of CD4+ and CD8+ T cells, combined with the increased production of IFN-γ and TNF-α (13). Furthermore, Goding et al. found that dual blockade of PD1 and LAG3 induced obvious tumor regression in a B16-F10 model with recurrent melanoma (56).

## Clinical Evidence for the LAG3 Immunotherapy Strategy

On the basis of the remarkable efficacy of LAG3 in basic research models, clinical applications of LAG3 inhibitors have received considerable attention. Current targeting strategies in the clinic and the involvement between LAG3 and PD-1/PD-L1 are shown in **Figure 2**. By the end of 2019, clinical trials of 13 drugs targeting LAG3—one LAG3 fusion protein and 12 LAG3 inhibitors—as anticancer drugs were recruiting participants (data source: https://www.clinicaltrials.gov). A LAG3-immunoglobulin fusion protein termed IMP321 (Prima BioMed/Immutep) is the first LAG3 fusion protein to enter clinical trials. IMP321 is a soluble dimeric recombinant protein comprising four LAG3 extracellular domains that bind to MHC-II and activate antigen presenting cells, including monocytes and DCs (57). Thirteen therapeutics currently in clinical and preclinical trials have focused on blockade with antagonistic mAbs by LAG3-targeting strategies. The first antagonistic mAb to LAG3 to enter the clinic was relatlimab. In a phase I/II study assessing the tolerability of relatlimab in combination with nivolumab, the objective response rate (ORR) was observed in 11.5% of patients with advanced melanoma whose tumors had progressed on previous anti-PD-1 or anti-PD-L1 immunotherapy (58). Furthermore, the ORR was more than three times higher in patients with TILs expressing LAG3 (>1%; 18% ORR) than in LAG3-negative patients (<1%; 5% ORR), irrespective of PD-L1 status. In addition, a phase I clinical trial of IMP321 for the treatment of advanced renal cell carcinoma and advanced pancreatic cancer was successfully completed (59). Brignone et al. conducted a phase I/II trial of IMP321 combined with paclitaxel for the treatment of metastatic breast cancer in 2010; their results demonstrated a 50% objective response rate (60). Active Immunotherapy PAClitaxel (AIPAC) is a double blind, placebo-controlled, randomized Phase IIb trial initiated in 2015, which enrolled 211 patients with metastatic hormone receptor-positive (HR+) breast cancer ineligible for HER-2/neu therapy to evaluate the safety and efficacy of IMP321 added to weekly paclitaxel as a first-line chemotherapy versus paclitaxel plus placebo (61) (clinicaltrials.gov trial no. NCT02614833). This trial administered weekly paclitaxel (80 mg/m2 iv. day 1, 8 and 15 every 4 weeks) and consisted of two parts: (1) stage 1, two dose levels of IMP321 (6 and 30 mg sc.) were investigated to confirm the recommended Phase II dose of IMP321 in combination with weekly paclitaxel; (2) stage 2, patients were randomized 1:1 to receive paclitaxel plus IMP321 or paclitaxel plus placebo. The primary end point for stage 1 was to determine DLTs and the recommended Phase II dose. The secondary end points were OS, AEs, the time to next treatment, and the objective response rate (61). Multiple clinical trials have also explored the use of IMP321 as a novel immunologic adjuvant for advanced melanoma. IMP321 plus Montanide ISA51 VG (mannide monooleate surfactant and mineral oil) was combined with various tumor-specific peptides to activate tumor-specific CD8+ T cells and induce helper CD4+ T-cell responses (62). In a separate phase I trial, Romano et al. combined IMP321 with melanoma antigen recognized by T-cells 1 (MART-1) for the treatment of advanced melanoma. Although no therapeutic effect was observed, on the basis of the criteria for the evaluation of solid tumors, the number of MART-1-specific CD8+ T cells was significantly increased in the treatment group (63). The immunological and clinical outcomes of IMP321 therapy have varied among trials. Some of these differences may be attributable to trial design and the complex diversity in biology and tumor histology. However, IMP321 exhibited promising evidence of activity in patients with metastatic breast cancer (a weakly immunogenic type of tumor).

BMS-986016, developed by Bristol Myers Squibb, was the first humanized IgG4 anti-LAG3 monoclonal antibody. It is currently undergoing evaluation in several phase I/II trials, in combination with anti-PD-1/PD-L1, for the treatment of various solid tumors and hematological malignancies; to date, outcomes have been encouraging. Among patients with melanoma for whom anti-PD-1/PD-L1 treatment was ineffective (clinicaltrials.gov trial no. NCT01968109), the combination of BMS-986016 and an anti-PD-1 agent (nivolumab) demonstrated a good effect with safety similar to that of nivolumab monotherapy. LAG525, developed by Novartis, was the second humanized IgG4 anti-LAG3 monoclonal antibody, which is also in phase I/II clinical trials to assess its safety and pharmacokinetic profile. In this trial, LAG525 was administered as monotherapy or in combination with PDR001 (a novel anti-PD1 inhibitor) (clinicaltrials.gov trial no. NCT02460224). Other trials reported responses of combination LAG525 (anti-LAG3) and spartalizumab (anti-PD-1) in 12 of 121 (9.9%) patients with solid malignancies, including 2 of 8 (25%) patients with mesothelioma and 2 of 5 (40%) patients with triple negative breast cancer.

Dual blockade of LAG3 and PD-1 might enhance anti-tumor immunity in a synergistic manner. Therefore, bispecific anti-LAG3/PD-1 targeted drugs with broad utility have been developed for clinical applications. MacroGenics developed a bispecific drug MGD013, which is synthesized by Dual-Affinity Re-Targeting^®^. MGD013 simultaneously targets LAG3 and PD-1, thereby blocking immune checkpoint inhibition, activating T cells, and enhancing anti-tumor immunity. In addition, F-star developed a bispecific antibody drug, FS118, synthesized by modular antibody technology, which simultaneously targets LAG3 and PD-L1. Following a phase I clinical trial (clinicaltrials.gov trial no. NCT03440437), in which the pharmacokinetics and activity of FS118 were measured to evaluate its safety and tolerance, several clinical trials have begun active recruitment. All ongoing clinical trials that focus on the synergistic actions of LAG3 and PD-1/PD-L1 inhibitors are shown in **Table S1**.

## Discussion

On the basis of preclinical or clinical evidence supporting its promising synergistic effects when combined with PD-1/PD-L1 blockade, inhibition of LAG3 will presumably play an increasingly important role in anti-tumor immunotherapy. LAG3 is an immunoglobulin superfamily member that helps to maintain homeostasis in the immune system. Based on promising pre-clinical human and murine studies with LAG3-targeted therapies in combination with anti-PD-1/PD-L1, several clinical studies are ongoing to fully evaluate their safety and efficacy. Previous studies have demonstrated that chronic inflammatory environments (e.g., chronic viral infection or tumors) result in sustained T-cell activation, which causes persistent LAG3 expression on T cells. Notably, LAG3 is frequently coexpressed with other inhibitory receptors (e.g., PD1, TIGIT, TIM3, 2B4, and CD160), finally resulting in a T-cell dysfunctional state. However, there has been minimal research concerning the synergistic interactions of LAG3 with other promising inhibitory receptors, such as cytotoxic T lymphocyte associated antigen-4 and TIM3; these interactions should be investigated in future studies.

## Author Contributions

Conception and design: YQ, QL. Literature search: YQ, LC, QL. Writing, review, and/or revision of the manuscript: YQ, LC, XK. Guidance for the literature search process: JW, YF. All authors contributed to the article and approved the submitted version.

## Funding

This work was supported by the Beijing Municipal Natural Science Foundation (No. 7204293 and No. 7191009), the Special Research Fund for Central Universities, Peking Union Medical College (No. 3332019053), the Beijing Hope Run Special Fund of Cancer Foundation of China (No. LC2019B03 and No. LC2019L07), the Natural Science Foundation of China (No. 81872160), and National Key R&D Program of China (No. 2018YFC1315000 and No. 2018YFC1315003). 

## Conflict of Interest

The authors declare that the research was conducted in the absence of any commercial or financial relationships that could be construed as a potential conflict of interest.

## References

[1] Bertucci F Gonçalves A Immunotherapy in Breast Cancer: the Emerging Role of PD-1 and PD-L1. Curr Oncol Rep (2017) 19:64. 10.1007/s11912-017-0627-0 28799073

[2] Wang X Qi Y Kong X Zhai J Li Y Song Y Immunological therapy: A novel thriving area for triple-negative breast cancer treatment. Cancer Lett (2019) 442:409–28. 10.1016/j.canlet.2018.10.042 30419345

[3] Wolchok JD Chiarion-Sileni V Gonzalez R Rutkowski P Grob JJ Cowey CL Overall Survival with Combined Nivolumab and Ipilimumab in Advanced Melanoma. N Engl J Med (2017) 377:1345–56. 10.1056/NEJMoa1709684 PMC5706778 28889792

[4] Bellmunt J de Wit R Vaughn DJ Fradet Y Lee JL Fong L Pembrolizumab as Second-Line Therapy for Advanced Urothelial Carcinoma. N Engl J Med (2017) 376:1015–26. 10.1056/NEJMoa1613683 PMC5635424 28212060

[5] Ferris RL Blumenschein G Fayette J Guigay J Colevas AD Licitra L Nivolumab for Recurrent Squamous-Cell Carcinoma of the Head and Neck. N Engl J Med (2016) 375:1856–67. 10.1056/NEJMoa1602252 PMC5564292 27718784

[6] Nghiem PT Bhatia S Lipson EJ Kudchadkar RR Miller NJ Annamalai L PD-1 Blockade with Pembrolizumab in Advanced Merkel-Cell Carcinoma. N Engl J Med (2016) 374:2542–52. 10.1056/NEJMoa1603702 PMC4927341 27093365

[7] Brahmer J Reckamp KL Baas P Crinò L Eberhardt WE Poddubskaya E Nivolumab versus Docetaxel in Advanced Squamous-Cell Non-Small-Cell Lung Cancer. N Engl J Med (2015) 373:123–35. 10.1056/NEJMoa1504627 PMC4681400 26028407

[8] Motzer RJ Escudier B McDermott DF George S Hammers HJ Srinivas S Nivolumab versus Everolimus in Advanced Renal-Cell Carcinoma. N Engl J Med (2015) 373:1803–13. 10.1056/NEJMoa1510665 PMC5719487 26406148

[9] Andrews LP Marciscano AE Drake CG Vignali DA LAG3 (CD223) as a cancer immunotherapy target. Immunol Rev (2017) 276:80–96. 10.1111/imr.12519 28258692 PMC5338468

[10] Turnis ME Andrews LP Vignali DA Inhibitory receptors as targets for cancer immunotherapy. Eur J Immunol (2015) 45:1892–905. 10.1002/eji.201344413 PMC4549156 26018646

[11] Sun C Mezzadra R Schumacher TN Regulation and Function of the PD-L1 Checkpoint. Immunity (2018) 48:434–52. 10.1016/j.immuni.2018.03.014 PMC7116507 29562194

[12] Ishida Y Agata Y Shibahara K Honjo T Induced expression of PD-1, a novel member of the immunoglobulin gene superfamily, upon programmed cell death. EMBO J (1992) 11:3887–95. 10.1002/j.1460-2075.1992.tb05481.x PMC556898 1396582

[13] Huang R-Y Cheryl E Shashikant L Protul S Junko M Kunle O LAG3 and PD1 co-inhibitory molecules collaborate to limit CD8+ T cell signaling and dampen antitumor immunity in a murine ovarian cancer model. Oncotarget (2015) 6:27359–77. 10.18632/oncotarget.4751 PMC4694995 26318293

[14] Dong H Zhu G Tamada K Chen L B7-H1, a third member of the B7 family, co-stimulates T-cell proliferation and interleukin-10 secretion. Nat Med (1999) 5:1365–9. 10.1038/70932 10581077

[15] Keir ME Liang SC Guleria I Latchman YE Qipo A Albacker LA Tissue expression of PD-L1 mediates peripheral T cell tolerance. J Exp Med (2006) 203:883–95. 10.1084/jem.20051776 PMC2118286 16606670

[16] Triebel F Jitsukawa S Baixeras E Roman-Roman S Genevee C Viegas-Pequignot E LAG-3, a novel lymphocyte activation gene closely related to CD4. J Exp Med (1990) 171:1393–405. 10.1084/jem.171.5.1393 PMC2187904 1692078

[17] Zhang YC Li J Zhan YL Wu LQ Yu XY Zhang WJ Analysis of serum cytokines in patients with severe acute respiratory syndrome. Infect Immun (2004) 72:4410–5. 10.1128/IAI.72.8.4410-4415.2004 PMC470699 15271897

[18] Workman CJ Dugger KJ Vignali DA Cutting edge: molecular analysis of the negative regulatory function of lymphocyte activation gene-3. J Immunol (Baltimore Md 1950) (2002) 169:5392–5. 10.4049/jimmunol.169.10.5392 12421911

[19] Workman CJ Vignali DA The CD4-related molecule, LAG-3 (CD223), regulates the expansion of activated T cells. Eur J Immunol (2003) 33:970–9. 10.1002/eji.200323382 12672063

[20] Dumic J Dabelic S Flögel M Galectin-3: an open-ended story. Biochim Biophys Acta (2006) 1760:616–35. 10.1016/j.bbagen.2005.12.020 16478649

[21] Kouo T Huang L Pucsek AB Cao M Solt S Armstrong T Galectin-3 Shapes Antitumor Immune Responses by Suppressing CD8+ T Cells via LAG-3 and Inhibiting Expansion of Plasmacytoid Dendritic Cells. Cancer Immunol Res (2015) 3:412–23. 10.1158/2326-6066.CIR-14-0150 PMC4390508 25691328

[22] Liu W Tang L Zhang G Wei H Cui Y Guo L Characterization of a novel C-type lectin-like gene, LSECtin: demonstration of carbohydrate binding and expression in sinusoidal endothelial cells of liver and lymph node. J Biol Chem (2004) 279:18748–58. 10.1074/jbc.M311227200 14711836

[23] Moss ML Minond D Recent Advances in ADAM17 Research: A Promising Target for Cancer and Inflammation. Mediators Inflamm (2017) 2017:9673537. 10.1155/2017/9673537 29230082 PMC5688260

[24] Mao X Ou MT Karuppagounder SS Kam TI Yin X Xiong Y Pathological α-synuclein transmission initiated by binding lymphocyte-activation gene 3. Science (New York NY) (2016) 353:aah3374. 10.1126/science.aah3374 PMC5510615 27708076

[25] Li N Workman CJ Martin SM Vignali DA Biochemical analysis of the regulatory T cell protein lymphocyte activation gene-3 (LAG-3; CD223). J Immunol (Baltimore Md 1950) (2004) 173:6806–12. 10.4049/jimmunol.173.11.6806 15557174

[26] Delmastro MM Styche AJ Trucco MM Workman CJ Vignali DAA Piganelli JD Modulation of redox balance leaves murine diabetogenic TH1 T cells “LAG-3-ing” behind. Diabetes (2012) 61:1760–8. 10.2337/db11-1591 PMC3379669 22586584

[27] Li N Wang Y Forbes K Vignali KM Heale BS Saftig P Metalloproteases regulate T-cell proliferation and effector function via LAG-3. EMBO J (2007) 26:494–504. 10.1038/sj.emboj.7601520 17245433 PMC1783452

[28] Avice MN Sarfati M Triebel F Delespesse G Demeure CE Lymphocyte activation gene-3, a MHC class II ligand expressed on activated T cells, stimulates TNF-alpha and IL-12 production by monocytes and dendritic cells. J Immunol (1999) 162:2748–53. 10072520

[29] Triebel F Hacene K Pichon M-F A soluble lymphocyte activation gene-3 (sLAG-3) protein as a prognostic factor in human breast cancer expressing estrogen or progesterone receptors. Cancer Lett (2006) 235:147–53. 10.1016/j.canlet.2005.04.015 15946792

[30] Li N Jilisihan B Wang W Tang Y Keyoumu S Soluble LAG3 acts as a potential prognostic marker of gastric cancer and its positive correlation with CD8+T cell frequency and secretion of IL-12 and INF-γ in peripheral blood. Cancer Biomark (2018) 23:341–51. 10.3233/CBM-181278 PMC13078572 30223387

[31] Camisaschi C Casati C Rini F Perego M De Filippo A Triebel F LAG-3 expression defines a subset of CD4(+)CD25(high)Foxp3(+) regulatory T cells that are expanded at tumor sites. J Immunol (Baltimore Md 1950) (2010) 184:6545–51. 10.4049/jimmunol.0903879 20421648

[32] Bae J Lee SJ Park CG Lee YS Chun T Trafficking of LAG-3 to the surface on activated T cells via its cytoplasmic domain and protein kinase C signaling. J Immunol (Baltimore Md 1950) (2014) 193:3101–12. 10.4049/jimmunol.1401025 25108024

[33] Salgado R Denkert C Demaria S Sirtaine N Klauschen F Pruneri G The evaluation of tumor-infiltrating lymphocytes (TILs) in breast cancer: recommendations by an International TILs Working Group 2014. Ann Oncol (2015) 26:259–71. 10.1093/annonc/mdu450 PMC6267863 25214542

[34] Baixeras E Huard B Miossec C Jitsukawa S Martin M Hercend T Characterization of the lymphocyte activation gene 3-encoded protein. A new ligand for human leukocyte antigen class II antigens. J Exp Med (1992) 176:327–37. 10.1084/jem.176.2.327 PMC2119326 1380059

[35] Solinas C Migliori E De Silva P Willard-Gallo K LAG3: The Biological Processes That Motivate Targeting This Immune Checkpoint Molecule in Human Cancer. Cancers (2019) 11:1213. 10.3390/cancers11081213 PMC6721578 31434339

[36] Maruhashi T Okazaki IM Sugiura D Takahashi S Maeda TK Shimizu K LAG-3 inhibits the activation of CD4 T cells that recognize stable pMHCII through its conformation-dependent recognition of pMHCII. Nat Immunol (2018) 19:1415–26. 10.1038/s41590-018-0217-9 30349037

[37] Kurachi M CD8 T cell exhaustion. Semin Immunopathol (2019) 41:327–37. 10.1007/s00281-019-00744-5 30989321

[38] Liang B Workman C Lee J Chew C Dale BM Colonna L Regulatory T cells inhibit dendritic cells by lymphocyte activation gene-3 engagement of MHC class II. J Immunol (Baltimore Md 1950) (2008) 180:5916–26. 10.4049/jimmunol.180.9.5916 18424711

[39] Workman CJ Rice DS Dugger KJ Kurschner C Vignali DA Phenotypic analysis of the murine CD4-related glycoprotein, CD223 (LAG-3). Eur J Immunol (2002) 32:2255–63. 10.1002/1521-4141(200208)32:8<2255::AID-IMMU2255>3.0.CO;2-A 12209638

[40] Ascierto PA Bono P Bhatia S Melero I Nyakas MS Svane IM LBA18Efficacy of BMS-986016, a monoclonal antibody that targets lymphocyte activation gene-3 (LAG-3), in combination with nivolumab in pts with melanoma who progressed during prior anti–PD-1/PD-L1 therapy (mel prior IO) in all-comer and biomarker-enriched populations. Ann Oncol (2017) 28:611–2. 10.1093/annonc/mdx440.011

[41] Workman CJ Vignali DAA Negative Regulation of T Cell Homeostasis by Lymphocyte Activation Gene-3 (CD223). J Immunol (2005) 174:688. 10.4049/jimmunol.174.2.688 15634887

[42] Blackburn SD Shin H Haining WN Zou T Workman CJ Polley A Coregulation of CD8+ T cell exhaustion by multiple inhibitory receptors during chronic viral infection. Nat Immunol (2009) 10:29–37. 10.1038/ni.1679 19043418 PMC2605166

[43] Woo SR Turnis ME Goldberg MV Bankoti J Selby M Nirschl CJ Immune inhibitory molecules LAG-3 and PD-1 synergistically regulate T-cell function to promote tumoral immune escape. Cancer Res (2012) 72:917–27. 10.1158/0008-5472.CAN-11-1620 PMC3288154 22186141

[44] Andrews LP Yano H Vignali DAA Inhibitory receptors and ligands beyond PD-1, PD-L1 and CTLA-4: breakthroughs or backups. Nat Immunol (2019) 20:1425–34. 10.1038/s41590-019-0512-0 31611702

[45] Rådestad E Klynning C Stikvoort A Mogensen O Nava S Magalhaes I Immune profiling and identification of prognostic immune-related risk factors in human ovarian cancer. Oncoimmunology (2019) 8:e1535730. 10.1080/2162402X.2018.1535730 30713791 PMC6343785

[46] Matsuzaki J Gnjatic S Mhawech-Fauceglia P Beck A Miller A Tsuji T Tumor-infiltrating NY-ESO-1-specific CD8+ T cells are negatively regulated by LAG-3 and PD-1 in human ovarian cancer. Proc Natl Acad Sci USA (2010) 107:7875–80. 10.1073/pnas.1003345107 PMC2867907 20385810

[47] Koch K Koch N Sandaradura de Silva U Jung N Schulze zur Wiesch J Fätkenheuer G Increased Frequency of CD49b/LAG-3(+) Type 1 Regulatory T Cells in HIV-Infected Individuals. AIDS Res Hum Retroviruses (2015) 31:1238–46. 10.1089/aid.2014.0356 26192268

[48] Hannier S Triebel F The MHC class II ligand lymphocyte activation gene-3 is co-distributed with CD8 and CD3-TCR molecules after their engagement by mAb or peptide-MHC class I complexes. Int Immunol (1999) 11:1745–52. 10.1093/intimm/11.11.1745 10545478

[49] Bruniquel D Borie N Hannier S Triebel F Regulation of expression of the human lymphocyte activation gene-3 (LAG-3) molecule, a ligand for MHC class II. Immunogenetics (1998) 48:116–24. 10.1007/s002510050411 9634475

[50] Maçon-Lemaître L Triebel F The negative regulatory function of the lymphocyte-activation gene-3 co-receptor (CD223) on human T cells. Immunology (2005) 115:170–8. 10.1111/j.1365-2567.2005.02145.x PMC1782137 15885122

[51] Hannier S Tournier M Bismuth G Triebel F CD3/TCR complex-associated lymphocyte activation gene-3 molecules inhibit CD3/TCR signaling. J Immunol (1998) 161:4058–65. 9780176

[52] Hu S Liu X Li T Li Z Hu F LAG3 (CD223) and autoimmunity: Emerging evidence. J Autoimmun (2020) 112:102504. 10.1016/j.jaut.2020.102504 32576412

[53] Buisson S Triebel F MHC class II engagement by its ligand LAG-3 (CD223) leads to a distinct pattern of chemokine and chemokine receptor expression by human dendritic cells. Vaccine (2003) 21:862–8. 10.1016/S0264-410X(02)00533-9 12547595

[54] Du H Yi Z Wang L Li Z Niu B Ren G The co-expression characteristics of LAG3 and PD-1 on the T cells of patients with breast cancer reveal a new therapeutic strategy. Int Immunopharmacol (2020) 78:106113. 10.1016/j.intimp.2019.106113 31841754

[55] Woo S-R Turnis ME Goldberg MV Bankoti J Selby M Nirschl CJ Immune Inhibitory Molecules LAG-3 and PD-1 Synergistically Regulate T-cell Function to Promote Tumoral Immune Escape. Cancer Res (2012) 72:917–27. 10.1158/0008-5472.CAN-11-1620 PMC3288154 22186141

[56] Goding SR Wilson KA Xie Y Harris KM Baxi A Akpinarli A Restoring Immune Function of Tumor-Specific CD4+ T Cells during Recurrence of Melanoma. J Immunol (2013) 190:4899–909. 10.4049/jimmunol.1300271 PMC3633733 23536636

[57] Fougeray S Brignone C Triebel F A soluble LAG-3 protein as an immunopotentiator for therapeutic vaccines: Preclinical evaluation of IMP321. Vaccine (2006) 24:0–5433. 10.1016/j.vaccine.2006.03.050 16621192

[58] Burugu S Gao D Leung S Chia SK Nielsen TO LAG-3+ tumor infiltrating lymphocytes in breast cancer: clinical correlates and association with PD-1/PD-L1+ tumors. Ann Oncol (2017) 28:2977–84. 10.1093/annonc/mdx557 29045526

[59] Brignone C Escudier B Grygar C Marcu M Triebel F A Phase I Pharmacokinetic and Biological Correlative Study of IMP321, a Novel MHC Class II Agonist, in Patients with Advanced Renal Cell Carcinoma. Clin Cancer Res (2009) 15:6225–31. 10.1158/1078-0432.CCR-09-0068 19755389

[60] Brignone C Gutierrez M Mefti F Brain E Jarcau R Cvitkovic F First-line chemoimmunotherapy in metastatic breast carcinoma: combination of paclitaxel and IMP321 (LAG-3Ig) enhances immune responses and antitumor activity. J Transl Med (2010) 8:71. 10.1186/1479-5876-8-71 20653948 PMC2920252

[61] Dirix L Triebel F AIPAC: a Phase IIb study of eftilagimod alpha (IMP321 or LAG-3Ig) added to weekly paclitaxel in patients with metastatic breast cancer. Future Oncol (2019) 15:1963–73. 10.2217/fon-2018-0807 30977393

[62] Legat A Maby-El Hajjami H Baumgaertner P Cagnon L Abed-Maillard S Geldhof C Vaccination with LAG-3Ig (IMP321) and peptides induces specific CD4 and CD8 T-cell responses in metastatic melanoma patients - report of a phase I/IIa clinical trial. Clin Cancer Res (2016) 22:1330–40. 10.1158/1078-0432.CCR-15-1212 26500235

[63] Romano E Michielin O Voelter V Laurent J Bichat H Stravodimou A MART-1 peptide vaccination plus IMP321 (LAG-3Ig fusion protein) in patients receiving autologous PBMCs after lymphodepletion: results of a Phase I trial. J Trans Med (2014) 12:97. 10.1186/1479-5876-12-97 PMC4021605 24726012

