# Supplementary material for: Research Progress Concerning Dual Blockade of Lymphocyte-Activation Gene 3 and Programmed Death-1/Programmed Death-1 Ligand-1 Blockade in Cancer Immunotherapy: Preclinical and Clinical Evidence of This Potentially More Effective Immunotherapy Strategy
Source: Front Immunol. 2021 Jan 8;11:563258. doi: 10.3389/fimmu.2020.563258 (PMC7820761; doi:10.3389/fimmu.2020.563258)
Supplement: Supplementary file 1 [file DataSheet_1.pdf]

Supplementary Material

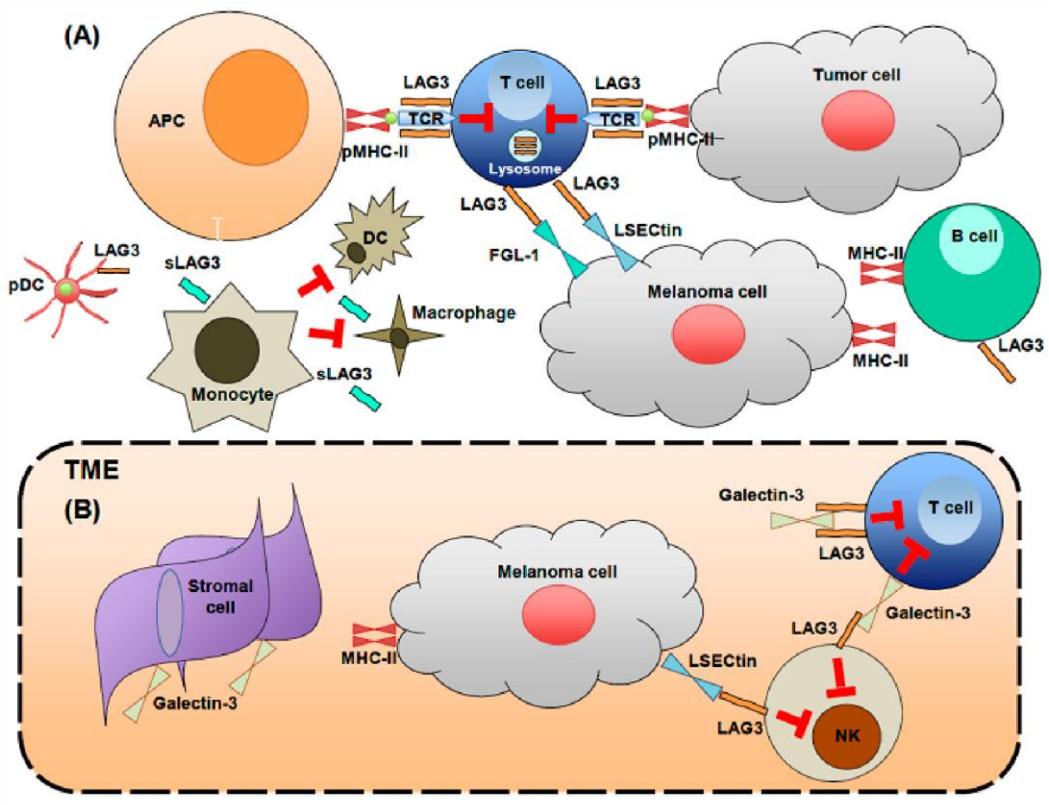

Figure S1. LAG3 biology on immune cells (A) and in the tumor microenvironment (TME) (B). (A) LAG3 is expressed on both CD4+ and CD8+ T cells, as well as on plasmacytoid dendritic cells (pDC); it is also expressed on natural killer (NK) cells in the TME (B). Its principle ligands include major histocompatibility complex class II (MHC-II), expressed on both antigen-presenting cells (APC) and tumor cells; liver sinusoidal endothelial cell lectin (LSECtin), expressed on melanoma cells; and galectin-3, expressed on some T cells and stromal cells in the TME (B). In its soluble form, LAG3 (sLAG3) impairs monocyte differentiation to dendritic cells (DC) and macrophages. In the TME, interactions mediated by LAG3 and its ligands are inhibitory (B). Reproduced with permission from [31]. Copyright © The Author(s). 2019. Reproduced with permission from the author(s). 2019 Licensee MDPI, open access.

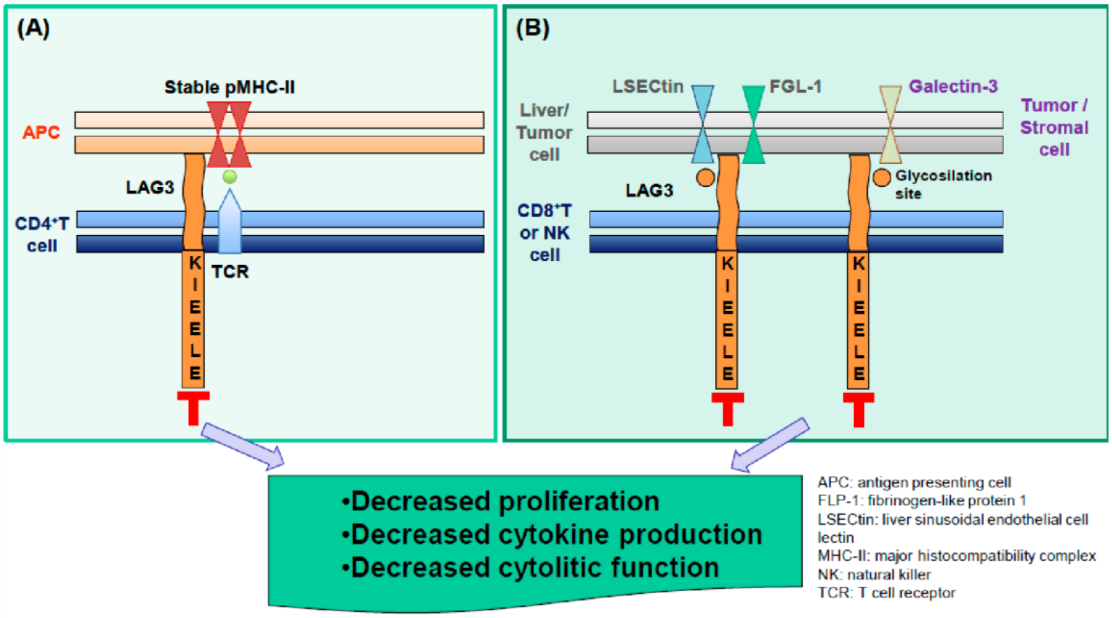

Figure S2 Molecular mechanisms of LAG3 function. LAG3 can be expressed on CD4+ T (A), CD8+ T, and NK cells (B). Interactions with its ligands (e.g., stable pMHC-II complexes, LSECtin, FGL-1, and/or galectin-3), expressed on different cells (i.e., immune, stromal, liver, and tumor cells), cause inhibition of CD4+, CD8+ and NK T-cell proliferation, as well as inhibition of cytokine production and cytolytic function. These effects are mediated by the “KIEELE” cytoplasmic motif of the LAG3 receptor. Reproduced with permission from [31]. Copyright © The Author(s). 2019. Reproduced with permission from the author(s). 2019 Licensee MDPI, open access.
