# Supplementary material for: Research Progress Concerning Dual Blockade of Lymphocyte-Activation Gene 3 and Programmed Death-1/Programmed Death-1 Ligand-1 Blockade in Cancer Immunotherapy: Preclinical and Clinical Evidence of This Potentially More Effective Immunotherapy Strategy
Source: Front Immunol. 2021 Jan 8;11:563258. doi: 10.3389/fimmu.2020.563258 (PMC7820761; doi:10.3389/fimmu.2020.563258)
Supplement: Supplementary file 2 [file Table_1.docx]

Table S1 Some ongoing clinical trials of anti-LAG3 combined with anti-PD-1/PD-L1 immunotherapeutic interventions

| No | Treatment ways | Target | NCT Number | Title | Status | Conditions | Interventions | Phase | Population | Study Start | Results First Posted |
| --- | --- | --- | --- | --- | --- | --- | --- | --- | --- | --- | --- |
| 1 | Monotherapy | Anti-PD-1; Anti-LAG-3; Anti-TIM-3 | NCT04370704 | Study of Combination Therapy With INCMGA00012 (Anti-PD-1), INCAGN02385 (Anti-LAG-3), and INCAGN02390 (Anti-TIM-3) in Participants With Select Advanced Malignancies | Not yet recruiting | Melanoma | Drug: INCAGN02385; Drug: INCAGN02390; Drug: INCMGA00012 | Phase 1; Phase 2 | 52 | 26-Jul-20 | No |
| 2 | Combination therapy | Anti-PD-1; Anti-LAG-3 | NCT04326257 | Personalized Immunotherapy in Patients With Recurrent /Metastatic SCCHN That Have Progressed on Prior Immunotherapy | Recruiting | Squamous Cell Carcinoma of the Head and Neck | Drug: Nivolumab+Relatlimab; Drug: Nivolumab+Ipilimumab | Phase 2 | 40 | May-20 | No |
| 3 | Combination therapy | Anti-PD-1; Anti-LAG-3 | NCT04140500 | Dose Escalation Study of a PD1- LAG3 Bispecific Antibody in Patients With Advanced and/or Metastatic Solid Tumors | Recruiting | Solid Tumors | Drug: RO7247669 | Phase 1 | 200 | 11-Nov-19 | No |
| 4 | Combination therapy | Anti-PD-1; Anti-LAG-3 | NCT04080804 | Study of Safety and Tolerability of Nivolumab Treatment Alone or in Combination With Relatlimab or Ipilimumab in Head and Neck Cancer | Recruiting | Head and Neck Squamous Cell Carcinoma (HNSCC) | Drug: Nivolumab; Drug: Relatlimab; Drug: Ipilimumab | Phase 2 | 60 | 22-Oct-19 | No |
| 5 | Combination therapy | Anti-PD-1; Anti-LAG-3 | NCT03743766 | Nivolumab, BMS-936558 in Combination With Relatlimab, BMS-986016 in Patients With Metastatic Melanoma Naïve to Prior Immunotherapy in the Metastatic Setting | Recruiting | Melanoma | Drug: Relatlimab; Drug: Nivolumab; Drug: Relatlimab + Nivolumab | Phase 2 | 42 | 29-Mar-19 | No |
| 6 | Monotherapy | Anti-PD-1; Anti-LAG-3 | NCT03642067 | Study of Nivolumab and Relatlimab in Patients With Microsatellite Stable (MSS) Advanced Colorectal Cancer | Recruiting | Microsatellite Stable (MSS) Colorectal Adenocarcinomas; Colorectal Adenocarcinoma | Drug: Nivolumab; Drug: Relatlimab | Phase 2 | 64 | 11-Feb-19 | No |
| 7 | Monotherapy | Anti-PD-1; Anti-LAG-3 | NCT03625323 | Combination Study With Soluble LAG-3 Fusion Protein Eftilagimod Alpha (IMP321) and Pembrolizumab in Patients With Previously Untreated Unresectable or Metastatic NSCLC, or Recurrent PD-X Refractory NSCLC or With Recurrent or Metastatic HNSCC | Recruiting | NSCLC; HNSCC | Drug: Eftilagimod alpha; Drug: Pembrolizumab | Phase 2 | 109 | 18-Feb-19 | No |
| 8 | Monotherapy | Anti-PD-1; Anti-LAG-3 | NCT03623854 | Nivolumab and Relatlimab in Treating Participants With Advanced Chordoma | Recruiting | Chordoma; Locally Advanced Chordoma; Metastatic Chordoma; Unresectable Chordoma | Biological: Nivolumab; Biological: Relatlimab | Phase 2 | 20 | 3-Apr-19 | No |
| 9 | Monotherapy | Anti-PD-1; Anti-LAG-3 | NCT03610711 | REACTION (Radiation Enhanced Assessment of Combination Therapies in Immuno-ONcology)- Nivolumab or Nivolumab in Combination With Other Immuno- oncology (IO) Agents After Targeted Systemic Radiation in Patients With Advanced Esophagogastric Cancer | Recruiting | Gastroesophageal Cancer; Immune Checkpoint Inhibition | Drug: Nivolumab; Drug: Relatlimab | Phase 1; Phase 2 | 30 | 6-Mar-19 | No |
| 10 | Monotherapy | Anti-PD-1; Anti-LAG-3 | NCT03607890 | Study of Nivolumab and Relatlimab in Advanced Mismatch Repair Deficient Cancers Resistant to Prior PD-(L)1 Inhibitor | Recruiting | Refractory MSI - H Solid Tumors Prior of PD-(L) 1 Therapy; MSI-H Tumors | Drug: Nivolumab; Drug: Relatlimab | Phase 2 | 21 | 14-Nov-18 | No |
| 11 | Monotherapy | Anti-PD-1; Anti-LAG-3 | NCT03493932 | Cytokine Microdialysis for Real- Time Immune Monitoring in Glioblastoma Patients Undergoing Checkpoint Blockade | Recruiting | Glioblastoma | Drug: Nivolumab; Drug: BMS-986016 | Phase 1 | 25 | 24-Sep-18 | No |
| 12 | Monotherapy | Anti-PD-1; Anti-LAG-3 | NCT03459222 | An Investigational Study of Immunotherapy Combinations in Participants With Solid Cancers That Are Advanced or Have Spread | Recruiting | Advanced Cancer | Biological: Relatlimab; Biological: Nivolumab; Drug: BMS-986205; Biological: Ipilimumab | Phase 1; Phase 2 | 230 | 25-May-18 | No |
| 13 | Combination therapy | Anti-PD-1; Anti-LAG-3 | NCT03440437 | FS118 First in Human Study in Patients With Advanced Malignancies After PD-1/PD-L1 Containing Therapy | Active, not recruiting | Advanced Cancer; Metastatic Cancer | Drug: FS118 | Phase 1 | 43 | 16-Apr-18 | No |
| 14 | Combination therapy | Anti-PD-1; Anti-LAG-3 | NCT03365791 | PDR001 Plus LAG525 for Patients With Advanced Solid and Hematologic Malignancies | Active, not recruiting | Small Cell Lung Cancer; Gastric Adenocarcinoma; Esophageal Adenocarcinoma; Castration Resistant Prostate Adenocarcinoma; Soft Tissue Sarcoma; Ovarian Adenocarcinoma; Advanced Well- differentiated Neuroendocrine Tumors; Diffuse Large B Cell Lymphoma | Biological: PDR001; Biological: LAG525 | Phase 2 | 76 | 24-Jan-18 | No |
| 15 | Monotherapy | Anti-PD-1; Anti-LAG-3 | NCT03250832 | Study of TSR-033 With an Anti- PD-1 in Patients With Advanced Solid Tumors | Recruiting | Advanced Solid Tumors; Antibodies; Immunotherapy; Colorectal Cancer | Drug: TSR-033; Drug: Anti-PD-1 | Phase 1 | 200 | 1-Aug-17 | No |
| 16 | Combination therapy | Anti-PD-1; Anti-LAG-3 | NCT03044613 | Nivolumab +/- Relatlimab Prior to Chemoradiation With II/III Gastro/ Esophageal Cancer | Recruiting | Gastric Cancer; Esophageal Cancer; GastroEsophageal Cancer | Drug: Nivolumab; Drug: Relatlimab; Drug: Carboplatin; Drug: Paclitaxel; Radiation: Radiation | Phase 1 | 25 | 11-Jul-17 | No |
| 17 | Monotherapy | Anti-PD-1; Anti-LAG-3 | NCT03005782 | Study of REGN3767 (Anti-LAG-3) With or Without REGN2810 (Anti- PD1) in Advanced Cancers | Recruiting | Malignancies | Drug: REGN3767; Drug: cemiplimab | Phase 1 | 669 | 7-Nov-16 | No |
| 18 | Combination therapy | Anti-PD-1; Anti-LAG-3 | NCT02966548 | Safety Study of BMS-986016 With or Without Nivolumab in Patients With Advanced Solid Tumors | Active, not recruiting | Cancer | Drug: Relatlimab; Drug: Nivolumab | Phase 1 | 45 | 28-Nov-16 | No |
| 19 | Monotherapy | Anti-PD-1; Anti-LAG-3 | NCT02817633 | A Phase 1 Study of TSR-022, an Anti-TIM-3 Monoclonal Antibody, in Patients With Advanced Solid Tumors (AMBER) | Recruiting | Advanced or Metastatic Solid Tumors | Drug: TSR-022; Drug: TSR-042, an anti-PD-1 antibody; Drug: TSR-033, an anti-LAG-3 antibody | Phase 1 | 873 | Jul-16 | No |
| 20 | Monotherapy | Anti-PD-1; Anti-LAG-3 | NCT02676869 | Phase 1 Study of IMP321 (Eftilagimod Alpha) Adjuvant to Anti-PD-1 Therapy in Unresectable or Metastatic Melanoma | Completed | Stage IV Melanoma; Stage III Melanoma | Drug: IMP321 (eftilagimod alpha); Drug: Pembrolizumab | Phase 1 | 24 | Apr-16 | No |
| 21 | Combination therapy | Anti-PD-1; Anti-LAG-3 | NCT02658981 | Anti-LAG-3 Alone & in Combination w/ Nivolumab Treating Patients w/ Recurrent GBM (Anti-CD137 Arm Closed 10/16/18) | Recruiting | Glioblastoma; Gliosarcoma; Recurrent Brain Neoplasm | Biological: Anti-LAG-3 Monoclonal Antibody BMS 986016; Biological: Anti-PD-1; Other: Pharmacological Study; Other: Laboratory Biomarker Analysis; Biological: Anti-CD137 | Phase 1 | 100 | Aug-16 | No |
| 22 | Combination therapy | Anti-PD-1; Anti-LAG-3 | NCT02460224 | Safety and Efficacy of LAG525 Single Agent and in Combination With PDR001 in Patients With Advanced Malignancies | Active, not recruiting | Advanced Solid Tumors | Drug: LAG525; Drug: PDR001 | Phase 1; Phase 2 | 490 | 17-Jun-15 | No |
| 23 | Combination therapy | Anti-PD-1; Anti-LAG-3 | NCT01968109 | An Investigational Immuno-therapy Study to Assess the Safety, Tolerability and Effectiveness of Anti-LAG-3 With and Without Anti- PD-1 in the Treatment of Solid Tumors | Recruiting | Neoplasms by Site | Biological: Relatlimab; Biological: Nivolumab; Biological: BMS-986213 | Phase 1; Phase 2 | 1500 | 14-Oct-13 | No |
